# A novel and efficient feature extraction algorithm using kmer-derived mutation signal

**DOI:** 10.7717/peerj.20940

**Published:** 2026-03-11

**Authors:** JingJing Zhang, XinGong Zhang, Jianwen Huang, RunBin Tang

**Affiliations:** School of Mathematical Sciences, Chongqing Normal University, Chongqing, China

**Keywords:** Phylogenetic analysis, Mutation signal simulation, Kmer, Weighted interval entropy

## Abstract

Effective algorithms for extracting genomic features are crucial for downstream bioinformatics research. Although kmer-based descriptive statistical features (frequency and position) offer a unique perspective for genomic feature extraction, their biological significance warrants further investigation. Therefore, uncovering the biological significance of kmers in genomes remains a significant challenge in current kmer-based genomic feature mining. In this work, based on reverse, complementary and reverse-complementary of kmers to simulate genomic accumulation mutation behavior, we proposed a novel feature extraction algorithm. By examining the elastic length region following each kmer for the presence of any of these three scenarios, kmers of potential biological significance are identified and defined them as anchors of sequence. To characterize cumulative information content of anchor on genome, we defined a weighted interval entropy, based on the interval signal of the identified anchors. In performance evaluation, we compared our proposed algorithm against methods based on position and frequency of kmer. Our method demonstrates superior effectiveness in species phylogenetic trees and machine learning classification. Furthermore, we conducted an in-depth analysis of the types and position of anchor, in Ebola-Zaire virus (EBOV) and hepatitis C virus (HCV) genomes of different sampling times, indicating that anchor can be used to diagnose the type and direction of accumulation of HCV and Ebola mutations.

## Introduction

Genome feature extraction is an important part of downstream bioinformatics research. When studying bioinformatics problems, efficient and concise genome features will make the constructed model interpretable and scalable. Extracting genome features based on continuous base fragments of k characters (kmer) can avoid the memory crisis and resource waste caused. The descriptive statistical characteristics of kmer in genomes have been widely used to extract genomic features, such as position (Ma et al., 2020) and frequency (Manekar & Sathe, 2018). Furthermore, the spacing signal of kmer in genome has also been used to explore uniqueness of genome. For example, by integrating spaced seeds into k-mers, a new and effective method for calculating genotypes was proposed (Häntze & Horton, 2023). PEER (Mitra, Bhattacharyya & Mukhopadhyay, 2020) mined feature of sequence based on waiting entropy of kmer, and conducted sequence classification research. KINN (Tang, Yu & Li, 2023) extracted features of sequence using internal distances between consecutive combinations of short characters to perform similarity comparisons between sequences.

Differences of molecular sequences are often directly due to internal and external environmental influences (Koonin, Dolja & Krupovic, 2022), such as the evolution of viruses. Virus is an important class of non-cellular organisms. Currently, viral species exhibit extremely high richness and diversity, driven by natural selection and directed mutation. In fact, once host is infected with virus, its immune system is activated to combat the invasion and suppress viral mutations. However, viruses employ targeted mutations to evade host immune responses, which in turn alter their genetic material (Koonin et al., 2020; Sanjuán & Domingo-Calap, 2016). Due to genomic mutations, structure of sequence is forced to change, leading to functional alterations in the mutated region (Domingo, 2016; Jia, Xu & Li, 2021), such as transmissibility, lethality and so on. Within the genome, mutations directly result in base duplications, insertions or deletions at the mutation site (Gordon & Halliday, 1995). Consequently, cumulative duplications of sequence segments are often found in genomes, and this tandem structure of sequence is used as a feature of sequence similarity, such as TRIM (Bhattacharyya, Mitra & Bhattacharyya, 2021). Furthermore, mutations break existing chemical bonds between bases, new chemical bonds are created to repair these bonds by indels of bases, which are also used to indicate tumor mutation behavior. For example, Ambigram (Li et al., 2023) simulated the progress of mutation and evolution of tumor, based on breakage-fusion-bridge events in the mutation process of tumor genomes.

In this work, we develop anchor, a novel genomic feature extraction algorithm, based on molecular cumulative mutation behavior and kmers, utilizing reverse complementary, reverse and self-copying of strings, to model different patterns of genomic mutational accumulation. In order to more effectively represent the cumulative mutation behaviors, we add the behaviors of insertion and deletion in the above several viral variants, *i.e*., an elastic region is added to the recognition process, which facilitates the recognition of more anchors. A kmer is identified as an anchor, if it possesses any of the three aforementioned properties within its subsequent elastic region. In fact, the number of anchors is much smaller than the number of kmers. To mitigate the problem of insufficient feature extraction due to a reduced number of anchors, we integrated the anchor interval signal entropy and defined penalty weight, based on its position in genome. In performance evaluation phase, we directly and indirectly tested the superior performance of the features on different viral dataset, by utilizing the based-anchor feature vectors to construct a phylogenetic tree and as input for machine learning, respectively. In our case study analysis, we selected the highly virulent Ebola pathogen and the common hepatitis C virus (HCV) virus to diagnostically profile their accumulated mutation patterns. By analyzing anchors in Ebola and HCV viruses of different types and time periods, we found that the continuous appearance and positional changes of anchors can be used to indicate the content and direction of accumulation of viral mutations.

## Materials and Methods

### Data sets

In this article, all viral sequences were obtained from NCBI (https://www.ncbi.nlm.nih.gov/). The size of the analysed dataset, the number of sequence types, and the average sequence length are presented in Table 1. The accession numbers for the sequences are detailed in the Supplemental Materials.

**Table 1:** Description of virus datasets used in the experiments.

Dataset ID	Dataset	Subtypes	Number	Sequence length
Dataset 1	Ebola	5	59	18,932
	HCV	6	82	9,427
Dataset 2	HIV	11	19,350	10,000
	HCV	5	2,791	10,000
	Dengue	4	156	10,827
	Influenza A	5	7,092	998–2,318

**Dataset 1:** For phylogenetic tree reconstruction, we investigated the relationship between sequence anchors and mutations in the hepatitis C virus (HCV) and the Ebola virus. HCV, classified into genotypes 1–6, can lead to severe liver disease, including cirrhosis and hepatocellular carcinoma. Ebola virus comprises five species: Ebola-Zaire (EBOV), Sudan (SUDV), Bundibugyo (BDBV), Ta Forest (TAFV), and Reston (RESTV), with RESTV not pathogenic in humans. The data set has also been used in previous studies (Wang, Yu & Li, 2024; He et al., 2020). The detailed data can be found in Tables S1 through S4 within the Supplemental Materials.

**Dataset 2:** For machine learning analyses, we used a separate dataset as specified below. The detailed data can be found in Tables S9 through S1.

### Definition of anchor point

Suppose DNA sequence is represented by $S = {S_{1}}{S_{2}} \cdots {S_{L}}$, and *L* is the length of the sequence. Then for any $i \in \{ 1,2, \cdots ,L\}$, ${S_{i}} \in \sigma = \{ A,C,G,T\}$. The $kmers$ of a sequence can be obtained from retrieving the sequence, based on a fixed-length ( $k$) sliding window with a sliding step of 1. For a fixed $k$, there are ${\left| \sigma \right|^k}$ possible forms of $kmer$, theoretically. But in fact, a sequence of length *L* has at most $L-k + 1$ kinds of $kmer$. In the process of viral mutation, bases near the mutation site are duplicated and rearranged, and recorded in the genome (Li et al., 2023). Based on the copied and rearraged behavior, we categorize the mutation into three cases with *kmer*: reverse complement, reverse order and replication. Replication of *kmer* is occurrences of identical-length string (denoted as *ks*) after the *kmer*, when $ks = kmer$. Reverse order of *kmer* is that *ks* is completely reversing the order of *kmer*, *e.g*., the reverse order of *ATC* is *CTA*. The reverse complement of *kmer* is generated by reversing and pairing, according to the principle of strict complement (A–T, C–G), *e.g*., the reverse complement of *AAC* is *GTT*. If a *kmer* belongs to one of the three mutation types above, the *kmer* is defined as an anchor. For example, a fragment of sequence is ACCGGT, *kmer* = ACC is an anchor, when *k = 3*.

**Figure 1:**
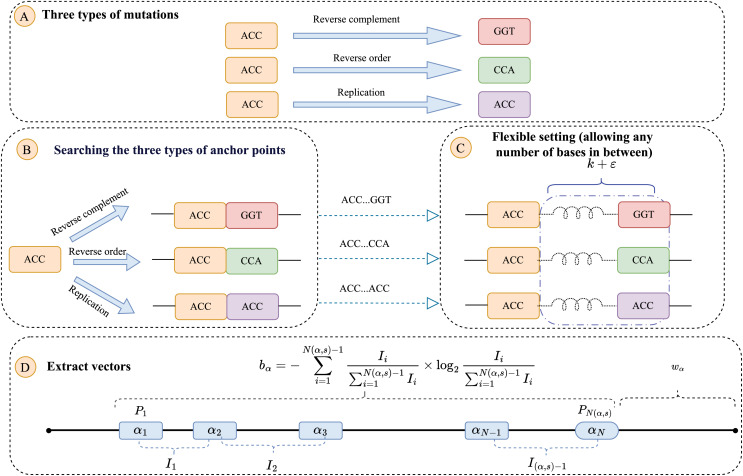
The flowchart of identifying anchor and the weighted interval entropy. Anchor selection flowchart. (A) Analyzing three types of mutations. (B) Three types of searching anchor points. (C) Adopting a flexible setting in the process of anchor recognition. (D) The weighted entropy $b{w_\alpha }$ for an anchor $\alpha$ was defined as ${w_\alpha } \times {b_\alpha }$, where ${b_\alpha }$ is the interval entropy and ${w_\alpha }$ is a weight based on the interval of the anchor.

In addition, insertion and deletion also occur frequently in virus mutation. So, we adopt a flexible setting in the process of anchor recognition. We increase the length of the region that identifies anchor. As shown in Fig. 1C, some bases can be inserted between *kmer* and *ks*. Anchors of sequence can be more recognized by increasing the elasticity setting, the parameter notation of the elasticity setting is denoted $\varepsilon$. So the length of region of recognizing anchor is less than or equal to $k + \varepsilon$ after *kmer*. Thus, elasticity setting is realistic in discovering small changes of sequence and conducting analysis of cumulative mutation in sequence.

### Sequence feature based on weighted interval entropy of anchor

Let $\alpha$ represent an anchor in sequence *S*. We denote the ordered occurrences of $\alpha$ in *S* as ${\alpha _{1}},{\alpha _{2}}, \cdots$, ${\alpha _{N(\alpha ,s)}}$, where $N(\alpha ,S)$ stands for the total number of occurrences of $\alpha$ in *S*, and we require $N(\alpha ,S) > 1$. Then ${\alpha _{i}}$ denotes the $i$-th occurrence of $\alpha$ in *S*. We denote ${P_\alpha }(S)$ as the position vector of $\alpha$ in *S*, ${P_\alpha }\left( S \right) = \left( {{P_{1}},{P_{2}}, \cdots ,{P_{N(\alpha ,s)}}} \right)$. Let ${I_\alpha }(S)$ be the interval vector between consecutive anchors, *i.e*.,

(1) $${I_\alpha }\left( S \right) = \left( {{I_{1}},{I_{2}}, \cdots ,{I_{N(\alpha ,S) - 1}}} \right) = \left( {{P_{2}} - {P_{1}}, \cdots ,{P_{N(\alpha ,s)}} - {P_{N(\alpha ,s) - 1}}} \right),$$

where ${I_{i}}$ is the *i*-th interval of the anchor point. Further normalize each interval, *i.e*.,

(2) $${p_{i,\alpha }} = {{{I_{i}}} \over {\sum\nolimits_{i = 1}^{N(\alpha ,s) - 1} {{I_{i}}} }},$$

Thus, the normalized vector of the anchor points $\alpha$ is

(3) $$p(\alpha ) = \left( {{p_{1,\alpha }},{p_{2,\alpha }}, \cdots ,{p_{N(\alpha ,s) - 1,\alpha }}} \right),$$

where $\sum\nolimits_{i = 1}^{N(\alpha ,s) - 1} {{p_{i,\alpha }}} = 1$. Finally, we use the Shannon entropy (Shannon, 1948) to define interval entropy of the anchor point $\alpha$ in the sequence *S*:

(4) $${b_\alpha } = - \sum\limits_{i = 1}^{N(\alpha ,s) - 1} {{p_{i,\alpha }}} \times {\log _{2}}{p_{i,\alpha }} = - \sum\limits_{i = 1}^{N(\alpha ,s) - 1} {{{{I_{i}}} \over {\sum\nolimits_{i = 1}^{N(\alpha ,s) - 1} {{I_{i}}} }}} \times {\log _{2}}{{{I_{i}}} \over {\sum\nolimits_{i = 1}^{N(\alpha ,s) - 1} {{I_{i}}} }}.$$

On the other hand, if the last position of anchor point is far from the end of the sequence, interval entropy of the anchor should be constrained. Therefore, we assign weight to the anchor interval entropy. The weight is defined as follows:

(5) $${w_\alpha } = {{L - {P_{N(\alpha ,s)}}} \over L} \times {\log _{2}}({{L - {P_{N(\alpha ,s)}}} \over L}),$$

If the anchor point is exactly at the end of the sequence, then $L - {P_{N(\alpha ,s)}} = 2k + \varepsilon$, for the general situation $L - {P_{N(\alpha ,s)}} \ge 2k + \varepsilon$, and so $L - {P_{N(\alpha ,s)}} \ne 0$. On the other hand, since $N(\alpha ,S) > 1$, then ${P_{N(\alpha ,s)}} \ne 0$, and then

(6) $${{L - {P_{N(\alpha ,s)}}} \over L}\lt1.$$

Anchor points located near the start or end of the sequence have smaller ${w_\alpha }$ values, indicating their relatively lower contribution. In other words, if the number of anchor points is abundant or scarce, its weighted interval entropy is low, which is consistent with the specificity of mutation. The weighted interval entropy $b{w_\alpha }$ of the anchor $\alpha$ is defined as follows:

(7) $$b{w_\alpha } = {w_\alpha } \times {b_\alpha },$$

$b{w_\alpha }$ is a quantitative indicator of the importance of the anchor point $\alpha$ within the entire sequence.

### Sequence numericalization based on anchor

For a sequence *S*, the weighted interval entropy of all anchors in sequence can be calculated based on weighted interval entropy *bw* described above. However, not all anchor in *S* are identified as anchors in other sequences, so the *bw* value of kmer, which is not considered anchors in other sequence, is defined as 0. Suppose there are *n* anchor types in the dataset. The *kmer* are sorted alphabetically and mapped to integers from 1 to *n*, and the sequence *S* is transformed into a numerical sequence *V*:

(8) $$V = \left( {b{w_{1}},b{w_{2}}, \cdots ,b{w_{n}}} \right).$$

### Parameter selection

When extracting anchors, the length of *kmer* and the elasticity length $\varepsilon$ need to be set in advance. If the parameter *k* is too large (or too small), the number of occurrences of *kmer* will be relatively small (or large). Moreover, according to the definition of anchor, *k* should not be set too large. So, we define the total contribution value of the anchor $H(k,\varepsilon )$, to fine-tune two parameters.

(9) $$H(k,\varepsilon ) = \sum\limits_{i}^{{4^k}} b {w_{i}}.$$

**Figure 2:**
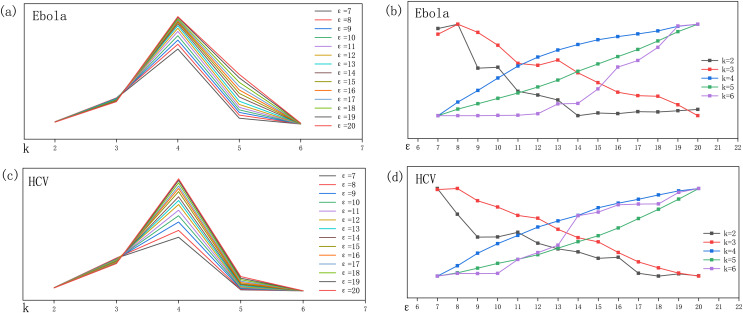
(A–D) Trend chart of the control variables for kmer length and elastic region length. The relationship between parameters k, $\varepsilon$, and the total entropy value.

Then, the relationship between $H(k,\varepsilon )$ and the two parameters is discussed separately, based on the idea of control variate. We construct the relationship diagram (Fig. 2) between variation of the total entropy value and parameter, in all sequences of Ebola and HCV viruses, respectively. In Figs. 2A and 2C, by controlling the value of *k*, it is found that $H(k,\varepsilon )$ is maximum when $k = 4$. In Figs. 2B and 2D, by controlling the size of $\varepsilon$, the trend of $H(k,\varepsilon )$ when $k \le 3$ is opposite to that of *H* when $k > 3$. It is caused with the number of anchor points: when *k* is small, the *kmers* appear more frequently; So when $\varepsilon$ increase, the probability of *kmer* becomes anchor will greatly increased. Meanwhile, when the length of anchor $\alpha$ interval vector is larger, the ${w_\alpha }$ becomes small. So $H(k,\varepsilon )$ shows a decreasing trend when $k \le 3$. From Figs. 2B and 2D, we can observe that when $k \ge 4$, $H(k,\varepsilon )$ increases with the value of $\varepsilon$. When $k = 4$ in Fig. 2B, the slope of the curve is gradually flatter at $\varepsilon = 13$. Hence, we set the parameters *k* and $\varepsilon$ to 4 and 13, respectively.

## Results

### Phylogenetic analysis based on anchors

To evaluate the performance of our algorithm, we conducted phylogenetic tree analysis. Any two sequences, ${S_{1}},{S_{2}}$, can be converted into numerical vectors ${V_{1}},{V_{2}}$ of equal dimension proposed method. The cosine distance of ${V_{1}},{V_{2}}$ is then computed as a measure of dissimilarity:

(10) $$\cos \left( {{V_{1}},{V_{2}}} \right) = {{{V_{1}} \cdot {V_{2}}} \over {\Vert{V_{1}}\Vert \cdot \Vert{V_{2}}\Vert}},$$

where ${V_{1}} \cdot {V_{2}}$ denotes the dot product of ${V_{1}}$ and ${V_{2}}$, $\Vert{V_{1}}\Vert$ and $\Vert{V_{2}}\Vert$ are mode of ${V_{1}}$ and ${V_{2}}$, respectively.

In this work, phylogenetic analysis of HCV and Ebola in Dataset 1 were performed based on the sequence distance matrices respectively. The sequence distance matrix was used as input to Mega X (Kumar et al., 2018), and the phylogenetic tree was generated using the neighbor-joining method (NJ) (Saitou & Nei, 1987). An online tool, iTol (Letunic & Bork, 2021), was used to beautify the phylogenetic tree for a more friendly visual presentation.

**Figure 3:**
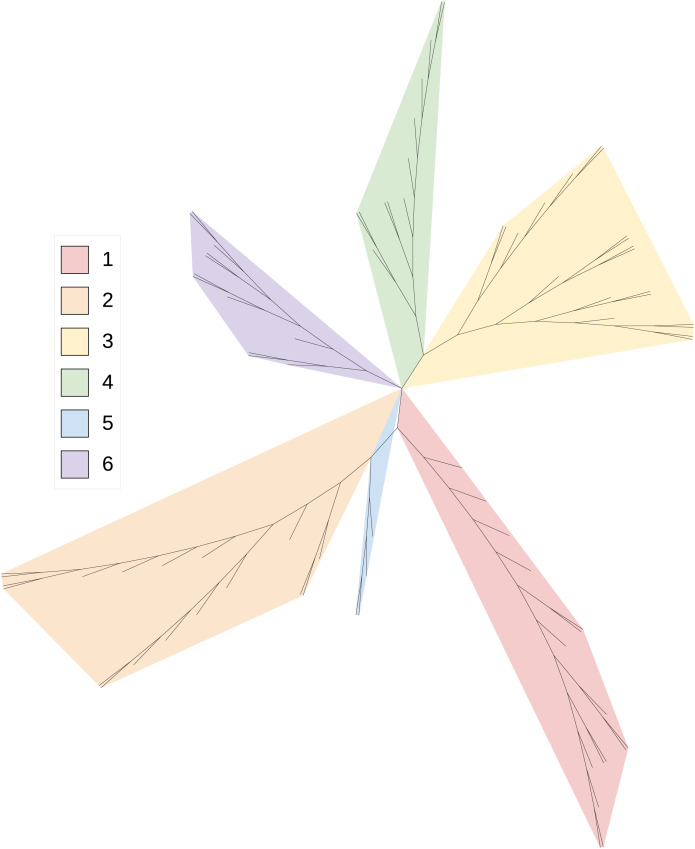
The result of phylogenetic analysis at HCV. Phylogenetic tree of HCV virus based on weighted interval entropy of anchor with parameters set to (4, 13).

A total of 82 HCV sequences were analyzed and the parameters $k,\varepsilon$ were set to 4, 13 respectively. The results are shown in Fig. 3, and all six subtype groups of HCV genome sequences are clustered distinctly and in different branches, and the six subtypes are also correctly classified.

**Figure 4:**
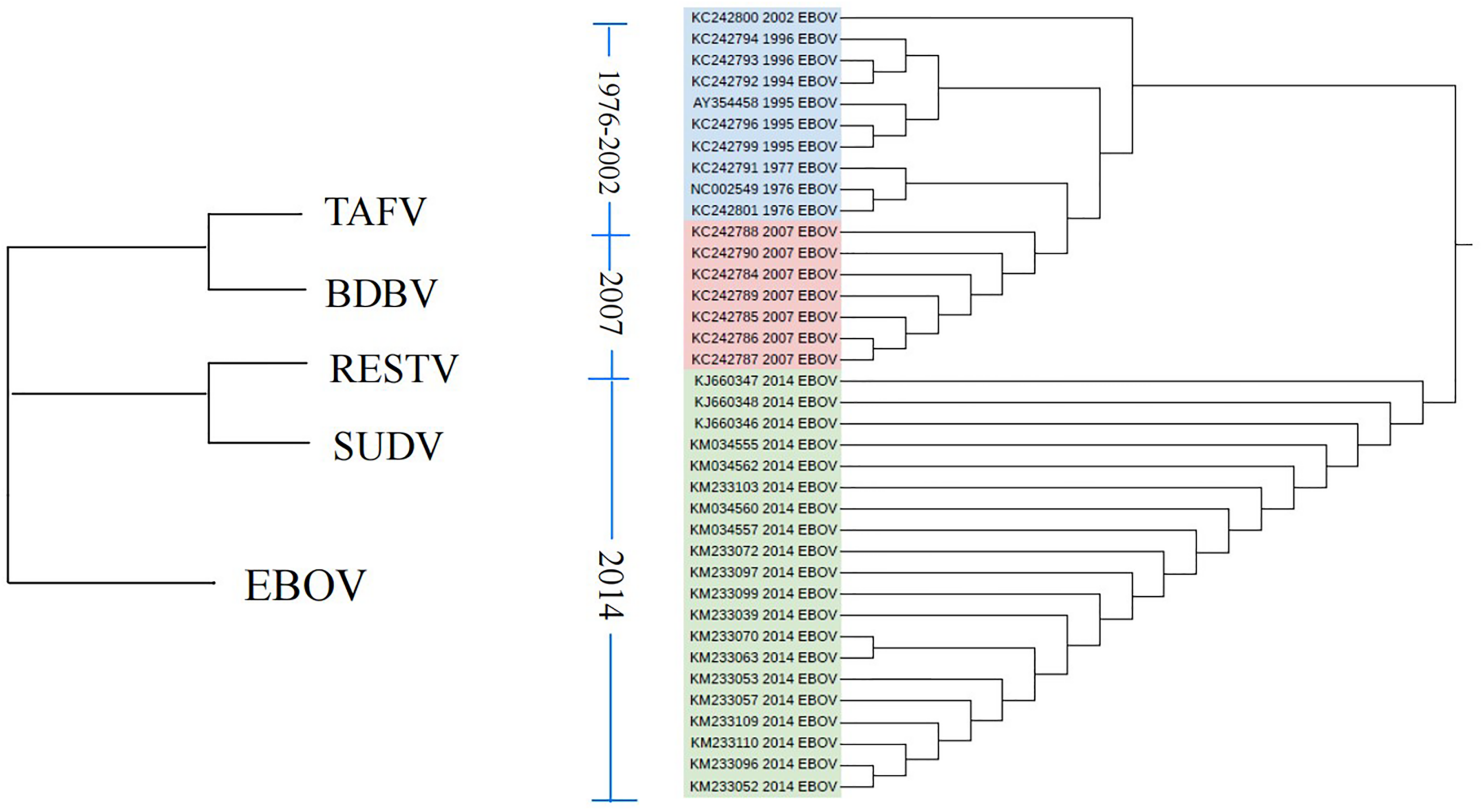
The result of phylogenetic analysis at Ebola. Phylogenetic tree of Ebola virus based on weighted interval entropy of anchor with parameters set to (4, 13).

When analyzing the classification problem for 59 Ebola sequences, the parameters $k,\varepsilon$ were also set to 4 and 13, respectively. The results, as shown in Fig. 4. The five types of Ebola virus can be clearly and efficiently distinguished from each other in the figure. In addition, the sequences in type EBOV, with three different collected time, can be clustered based on time. The above results suggest that the characterization of anchors can carve out some of the mutational order of the viral sequences.

### Performance evaluation based on machine learning

**Figure 5:**
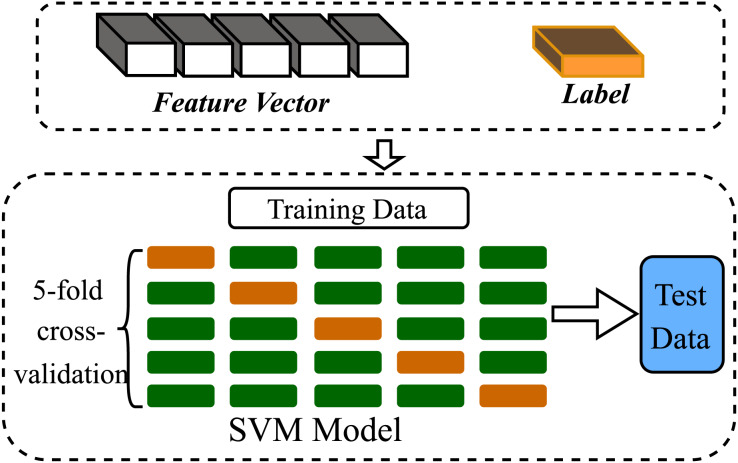
Machine learning (SVM) flowchart. The sequence feature vectors extracted by various alignment-free algorithms were used as input for machine learning, and supervised learning was performed.

To demonstrate the ability of feature vectors to express the uniqueness of sequences, we conducted performance evaluation of the feature extraction methods, based on the SVM algorithm in classification machine learning. In the experiments, we directly imported the SVM function from Scikit-learn library in Python 3.8. The model used an RBF kernel with parameters: C = 6, gamma = “scale”, class weight = “balanced”. We set decision function shape to “ovr”, random state to 42 and max_iter to 2,000 for reproducibility and convergence. All datasets were split into training and test sets in an 8:2 ratio, and all experiments were conducted on Intel Core i5-1135G7 Quad-Core Processor and 8 GB of RAM. During feature extraction, the parameter *k* was set to 4. The frequency feature of *kmers* were normalized before inputting. We performed five-fold cross-validation to minimize the variability in evaluation due to the randomness of dataset splitting. The experimental procedure is illustrated in Fig. 5.

#### Comparative experiment with partial feature of anchors

To evaluate the feature extraction capabilities of three types of patterns in kmer, we conducted separate comparative experiments on the three types of anchors. Such as each pattern independently, any combination of two patterns, and the fusion of all three patterns. As shown in Table 2, prediction accuracy of various combinations achieves high accuracy across all experimental settings. Furthermore, the performance differences among these approaches are marginal. More detailed comparison results are provided in Table S8 of Supplemental Material.

**Table 2:** Comparative experiment with partial feature anchors (Accuracy on Test Set Only).

Model	HIV	HCV	Dengue	Influenza A
All	0.9950	0.9964	**1.0000**	**0.9322**
Replication	0.9940	0.9982	**1.0000**	0.9268
Reverse complement	0.9935	0.9982	**1.0000**	0.9253
Reverse Order	**0.9961**	0.9982	**1.0000**	0.9230
Replication + Reverse order	0.9956	**1.0000**	**1.0000**	0.9268
Replication + Reverse complement	0.9953	0.9982	**1.0000**	**0.9322**
Reverse order + Reverse complement	0.9953	0.9964	**1.0000**	0.9299

**Note:**

The bold text indicates the maximum prediction accuracy across different feature combinations in the test set.

#### Performance comparison with existing method

From methodological perspective, the frequency feature of kmer considers only the occurrence counts of kmers, while position-weighted k-mer (Pwkmer) method emphasizes their positional information. Our proposed method incorporates information about frequency, position and spacing to ensure a more informative representation of the sequence. Therefore, we evaluated our feature extraction method by comparing it with FCGR (Hatje & Kollmar, 2012), BBC (Liu, Meng & Sun, 2008), RTD (Kolekar, Kale & Kulkarni-Kale, 2012), Pwkmer and KINN, based on frequency, position and correlation of kmer, respectively. Given that each genomic sequence is assigned to a distinct category, every dataset in Dataset 2 of this manuscript represents a multi-class classification problem. Accordingly, we use each sequence’s feature vector as the model input and its category as the prediction target, constituting a multi-class machine learning task.

In experiments, the feature vector extracted by Pwkmer, were multiplied by 1,000 to mitigate bias from genomic scale differences. The final results are presented in Table 3. Due to hardware configuration limitations, feature extraction from the Human Immunodeficiency Virus (HIV) dataset based on KINN method was restricted. Consequently, the corresponding prediction results are not provided.

**Table 3:** Performance comparison of different methods on virus sequence validation and test datasets.

Model	Metrics	HIV		HCV		Dengue		Influenza A	
		Train	Test	Train	Test	Train	Test	Train	Test
Anchor	Accuracy	**0.9947 $\pm$ 0.0006**	**0.9961**	0.9960 $\pm$ 0.0026	**1.0000**	0.9920 $\pm$ 0.0160	**1.0000**	0.9268 $\pm$ 0.0049	0.9322
	F1	**0.9767 $\pm$ 0.0066**	**0.9802**	0.9912 $\pm$ 0.0026	**1.0000**	0.9895 $\pm$ 0.0210	**1.0000**	0.9260 $\pm$ 0.0089	0.9377
	MCC	0.9913 $\pm$ 0.0010	**0.9935**	0.9930 $\pm$ 0.0045	**1.0000**	0.9893 $\pm$ 0.0213	**1.0000**	0.8998 $\pm$ 0.0069	0.9072
Kmer	Accuracy	0.9830 $\pm$ 0.0015	0.9830	0.9951 $\pm$ 0.0022	**1.0000**	**1.0000 $\pm$ 0.0000**	**1.0000**	0.9064 $\pm$ 0.0032	0.9129
	F1	0.9337 $\pm$ 0.0060	0.9331	0.9896 $\pm$ 0.0042	**1.0000**	**1.0000 $\pm$ 0.0000**	**1.0000**	0.9094 $\pm$ 0.0050	0.9200
	MCC	0.9719 $\pm$ 0.0024	0.9720	0.9915 $\pm$ 0.0038	**1.0000**	**1.0000 $\pm$ 0.0000**	**1.0000**	0.8724 $\pm$ 0.0045	0.8811
Pwkmer	Accuracy	0.9845 $\pm$ 0.0018	0.9807	0.9960 $\pm$ 0.0026	0.9980	**1.0000 $\pm$ 0.0000**	**1.0000**	0.9147 $\pm$ 0.0017	0.9183
	F1	0.9384 $\pm$ 0.0051	0.9248	0.9882 $\pm$ 0.0066	0.9890	**1.0000 $\pm$ 0.0000**	**1.0000**	0.9183 $\pm$ 0.0049	0.9280
	MCC	0.9745 $\pm$ 0.0029	0.9682	0.9930 $\pm$ 0.0045	0.9970	**1.0000 $\pm$ 0.0000**	**1.0000**	0.8835 $\pm$ 0.0023	0.8885
FCGR	Accuracy	0.9828 $\pm$ 0.0013	0.9833	0.9951 $\pm$ 0.0022	0.9950	**1.0000 $\pm$ 0.0000**	**1.0000**	0.9066 $\pm$ 0.0034	0.9129
	F1	0.9338 $\pm$ 0.0050	0.9325	0.9896 $\pm$ 0.0042	0.9950	**1.0000 $\pm$ 0.0000**	**1.0000**	0.9098 $\pm$ 0.0056	0.9198
	MCC	0.9717 $\pm$ 0.0022	0.9724	0.9915 $\pm$ 0.0038	0.9950	**1.0000 $\pm$ 0.0000**	**1.0000**	0.8727 $\pm$ 0.0047	0.8812
RTD	Accuracy	0.9664 $\pm$ 0.0025	0.9684	0.9937 $\pm$ 0.0039	**1.0000**	0.9920 $\pm$ 0.0160	0.9688	0.9318 $\pm$ 0.0035	0.9384
	F1	0.8592 $\pm$ 0.0146	0.8609	0.9841 $\pm$ 0.0096	**1.0000**	0.9902 $\pm$ 0.0195	0.9760	0.9304 $\pm$ 0.0050	0.9431
	MCC	0.9443 $\pm$ 0.0042	0.9477	0.9892 $\pm$ 0.0067	**1.0000**	0.9893 $\pm$ 0.0214	0.9581	0.9065 $\pm$ 0.0048	0.9154
BBC	Accuracy	0.6791 $\pm$ 0.0136	0.5459	0.9798 $\pm$ 0.0037	0.9911	**1.0000 $\pm$ 0.0000**	0.9688	0.8598 $\pm$ 0.0092	0.8760
	F1	0.4784 $\pm$ 0.0140	0.4442	0.9727 $\pm$ 0.0073	0.9946	**1.0000 $\pm$ 0.0000**	0.9699	0.8380 $\pm$ 0.0086	0.8568
	MCC	0.5858 $\pm$ 0.0126	0.4820	0.9651 $\pm$ 0.0065	0.9845	**1.0000 $\pm$ 0.0000**	0.9583	0.8094 $\pm$ 0.0121	0.8310
KINN	Accuracy	–	–	**0.9969 $\pm$ 0.0023**	**1.0000**	0.9920 $\pm$ 0.0160	**1.0000**	**0.9336 $\pm$ 0.0070**	**0.9407**
	F1	–	–	**0.9940 $\pm$ 0.0051**	**1.0000**	0.9902 $\pm$ 0.0195	**1.0000**	**0.9381 $\pm$ 0.0106**	**0.9386**
	MCC	–	–	**0.9946 $\pm$ 0.0040**	**1.0000**	0.9893 $\pm$ 0.0214	**1.0000**	**0.9092 $\pm$ 0.0092**	**0.9190**

**Note:**

- Due to device limitations, feature vector could not be successfully extracted. The bold text indicates the maximum values of different metrics among different models within the same validation and test dataset.

As shown in Table 3, the key performance metrics—Accuracy, F1 and MCC were evaluated on independent test sets for HIV, HCV, Dengue, and Influenza A. Through performance comparison on different datasets, our method demonstrated superior performance on among the HIV, HCV and Dengue datasets. On the Influenza A dataset, the performance of anchor is ranked third. In summary, anchor shows superior performance on datasets, particularly establishing a significant advantage in HIV virus recognition tasks.

### Relationship between sequence variation and anchors

To explore the association between anchor and viral mutations, we analyzed the differences in anchor points contained in the five virus types in Ebola virus dataset and contained in the six virus types in HCV dataset, respectively. By examining whether an anchor appears within a particular type, we found anchor point CGCG appeared only in BDBV type and all the sequences under BDBV type contained this anchor point (see Table 4). Further examination revealed that CGCG only occurs at positions 5,893 and 12,040 in the sequence, so it can be considered as a mutation unique, suggesting potential biological implications. Since the phylogenetic tree shows that RESTV and SUDV are relatively close to each other, we analyzed the anchors that are common and absent from the five types and their locations. As shown in the Table 4, in which the anchor points based on AACG, ACGG, ACGT, CGGT, CGTA, *etc*., we can easily observe that RESTV and SUDV contain anchor points, and the number and position of anchor points (see in Table S3) are relatively close to each other. We observe that RESTV and SUDV do not contain CCGC, CGCG, GCGT, while the other types contain such anchors. It could explain why these two type strains are relatively close.

**Table 4:** Information on the location of different types of anchors.

Type	TAFV	BDBV	RESTV	SUDV	EBOV
AACG	*	0	*	*	*
ACGG	*	0	*	*	*
ACGT	*	0	*	*	*
CGGT	0	0	*	*	*
CGTA	0	*	*	*	*
CCGC	*	0	0	0	*
CGCG	0	*	0	0	0
GCGT	0	0	0	0	*

**Note:**

* An anchor is present; more details results are available in Table S3.

Furthermore, we analyzed the anchor type and location of EBOV genome sequences from different sampling years (1994–1996, 2007); the result was shown in the Table 5. The results show that anchor CCCG only appears at the position 4,069 in 2007, and anchor GCGA only appears at the position 13,855 ( $\pm 8$) in the sequence collected in 2014.

**Table 5:** Anchor position information at different times in EBOV.

Type	1994	1995	1996	2007	2014
CCCG	0	0	0	4,069	0
GCGA	0	0	0	0	$13{,}\hbox855( \pm 8)$

Next, the differences of anchor in sequences at different time were analyzed in more detail, results are shown in Table 6 . In the table, ACGC appears at all times except 2007, so it is considered as the base anchor. We find the position of ACGC is unchanged from 1976 to 2002, but it is absent in 2007 and appears again in 2014. Therefore, it may be considered that it has not been naturally selected after mutation. In addition, CCTA is present in position 13,077 from 1976 in 2007, and is absent in 2014. Between 1994 and 1996, the number of anchors in Table S4 increased and the mutation occurred at roughly the same location. However, no anchor appears in 2014, one reason may be that drug inhibition changed the mutation direction of virus. We also find that GCGA is not occurring in 1976–1996, but appear firstly once in 2002. Finally, it is stably preserved at the position near 13,800 in 2014. We can easily observe the position of GTAC is 13,114 in 1976–2014. However, the number of GTAC again increased and stable existence in 2014.

**Table 6:** Anchor locations for different time portions of the sequence in EBOV.

Type	1976	1977	1994	1995	1996	2002	2007	2014
ACGC	*	*	*	*	*	*	0	*
CCTA	*	*	*	*	*	*	*	0
CGCA	*	*	*	*	*	*	0	*
CGTC	*	*	*	*	*	0	*	0
GCGA	0	0	0	0	0	*	0	*
GTAC	*	*	*	*	*	*	*	*
TCGC	*	*	*	*	*	*	0	*

**Note:**

* An anchor is present, more details results are available in Table S4.

In addition, to compare positions of anchors in six different types of HCV dataset, we selected one sequence from each type and analyzed its anchor sites. Ultimately, we discovered that, similar to Ebola virus, partial anchor sites differed across types. The anchor site is absent in type 5 but present in most other types, indicating this characteristic is not unique to Ebola virus. The detail results are available in Table S7.

Anchor points, with a sudden increase and stable positions, should be considered as accumulation mutation points with significance in the process of virus evolution. The sudden absence of anchor points may mean that the direction of virus evolution has changed. Thus, the anchor points may be important for determining the direction and type of mutations.

## Conclusions

In this study, we defined anchors based on molecular accumulation mutations behaviors, and proposed weighted interval entropy to quantify the information content of anchors in the genome. We used species phylogenetic trees and machine learning classification algorithms to directly and indirectly test the effectiveness of the anchor-based algorithm. Our analysis of the presence, position and number of genomic anchors revealed that they can serve as effective indicators for capturing common sequence features after mutations have accumulated. Our future research will focus on the relationship between anchors and the direction and path of viral accumulation mutations.

## Supplemental Information

10.7717/peerj.20940/supp-1 Supplemental Information 1 The information of sequences of HCV and Ebola. The position and type of anchor in sequence of HCV and Ebola.

## Additional Information and Declarations

### Data Availability

The following information was supplied regarding data availability:

The 26,742 sequences are available at NCBI (Table S1).

The code is available at GitHub and Zenodo:

- https://github.com/JingjingZhang111/anchor.

- JingjingZhang111. (2025). JingjingZhang111/anchor: This is the code for the a novel and efficient feature extraction algorithm using kmer-derived mutation signal (v1.1.0). Zenodo. https://doi.org/10.5281/zenodo.14732628.
